# Dynamic responses of picophytoplankton to physicochemical variation in the eastern Indian Ocean

**DOI:** 10.1002/ece3.5107

**Published:** 2019-04-01

**Authors:** Yuqiu Wei, Guicheng Zhang, Ju Chen, Jing Wang, Changling Ding, Xiaodong Zhang, Jun Sun

**Affiliations:** ^1^ Institute of Marine Science and Technology Shandong University Qingdao China; ^2^ College of Marine and Environmental Sciences Tianjin University of science and Technology Tianjin China; ^3^ Tianjin Key Laboratory of Marine Resources and Chemistry Tianjin University of Science and Technology Tianjin China; ^4^ State Key Laboratory of Tropical Oceanography (LTO) South China Sea Institute of Oceanology Chinese Academy of Sciences Guangzhou China

**Keywords:** dynamic response, eastern Indian Ocean, physicochemical condition, Picophytoplankton

## Abstract

Picophytoplankton were investigated during spring 2015 and 2016 extending from near‐shore coastal waters to oligotrophic open waters in the eastern Indian Ocean (EIO). They were typically composed of *Prochlorococcus *(*Pro*), *Synechococcus* (*Syn*), and picoeukaryotes (*PEuks*). *Pro* dominated most regions of the entire EIO and were approximately 1–2 orders of magnitude more abundant than *Syn* and *PEuks*. Under the influence of physicochemical conditions induced by annual variations of circulations and water masses, no coherent abundance and horizontal distributions of picophytoplankton were observed between spring 2015 and 2016. Although previous studies reported the limited effects of nutrients and heavy metals around coastal waters or upwelling zones could constrain *Pro* growth, *Pro* abundance showed strong positive correlation with nutrients, indicating the increase in nutrient availability particularly in the oligotrophic EIO could appreciably elevate their abundance. The exceptional appearance of picophytoplankton with high abundance along the equator appeared to be associated with the advection processes supported by the Wyrtki jets. For vertical patterns of picophytoplankton, a simple conceptual model was built based upon physicochemical parameters. However, *Pro* and *PEuks* simultaneously formed a subsurface maximum, while *Syn* generally restricted to the upper waters, significantly correlating with the combined effects of temperature, light, and nutrient availability. The average chlorophyll *a* concentrations (Chl *a*) of picophytoplankton accounted for above 49.6% and 44.9% of the total Chl *a* during both years, respectively, suggesting that picophytoplankton contributed a significant proportion of the phytoplankton community in the whole EIO.

## 1 INTRODUCTION

Three traditionally recognized groups of picophytoplankton (<3 µm), namely *Prochlorococcus *(*Pro*), *Synechococcus *(*Syn*), and picoeukaryotes (*PEuks*), constitute an essential component of phytoplankton community (Baer, Lomas, Terpis, Mouginot, & Martiny, 2017; Bertilsson, Berglund, Karl, & Chisholm, 2003; Worden & Not, 2008). *Pro* and *Syn* are the dominant and widespread members in the warm oligotrophic waters; however, *Pro* are normally more abundant than *Syn* in open oceans, often by 10 ~ fold or more (Flombaum et al., 2013; Johnson et al., 2006). In particular, *Syn* and *PEuks* all coexist in various environments, even from pole to pole (Chen et al., 2011). Observations in the oligotrophic Pacific Ocean and Atlantic Ocean showed that picophytoplankton contributed to approximately 60%–80% of the total marine primary productivity (Agustí & Llabrés, 2007; Campbell, Liu, Nolla, & Vaulot, 1997), suggesting that picophytoplankton has crucial roles in primary productivity in (sub)tropical oligotrophic regions (Matsumoto, Abe, Fujiki, Sukigara, & Mino, 2016). Collectively, owing to their high abundance, wide distribution, and large contribution to primary production, picophytoplankton have been known to have large impacts on marine ecosystem and biogeochemical cycles. Thus, presenting their biogeographic patterns is critical to understand different regional contributions to carbon cycle of these special taxa.

To date, many biostatistical models (such as quantitative niche models, parametric regression models, and neural network models) combining the variable characteristics of picophytoplankton and oceanic environments have been successfully constructed to simulate their biogeographic distributions (Flombaum et al., 2013). As one of the largest oligotrophic areas, however, the Indian Ocean (IO) particularly with respect to picophytoplankton has received far less attention than other oceans. Meanwhile, Clokie, Millard, Mehta, and Mann (2006) presented that picophytoplankton are the most abundant primary producers in the IO. Based on our size‐fractionated chlorophyll *a* analysis, indeed, picophytoplankton are responsible for a large fraction of phytoplankton community in eastern Indian Ocean (EIO), further confirming they are potentially important for the IO ecosystem and primary productivity. To enhance our appreciation of the importance of various forms of picophytoplankton, similar exercises should be conducted in regional scale with higher spatial resolution in the IO. The tropical IO forms the major part of the largest warm pool on the earth, and its interaction with the monsoon plays an important role in shaping complex circulation systems on both regional and global scales (Raven, 1998). In other words, surface circulations and water masses in the IO are considerably complex and highly variable because of its response to the annually reversing monsoon winds. Therefore, we speculated that the spatio‐temporal variability in picophytoplankton might be closely related to the annual variations of circulations or water masses in the EIO. We thereafter compared the picophytoplankton communities and associated environmental variables between spring 2015 and 2016 to address the following questions: (a) what are the different distributions of picophytoplankton abundance? (b) how do major environmental factors influence their abundances? and (c) what are different distributions as a result of the annual variations of circulations or water masses in the EIO?

In the present study, the dynamic responses of picophytoplankton communities to physicochemical variations associated with circulations or water masses were investigated by flow cytometry, to address the lacking data of picophytoplankton, and to understand the annual effects of environmental variables on picophytoplankton in the EIO.

## 2 MATERIALS AND METHODS

### 2.1 Sampling strategy

**Figure 1:**
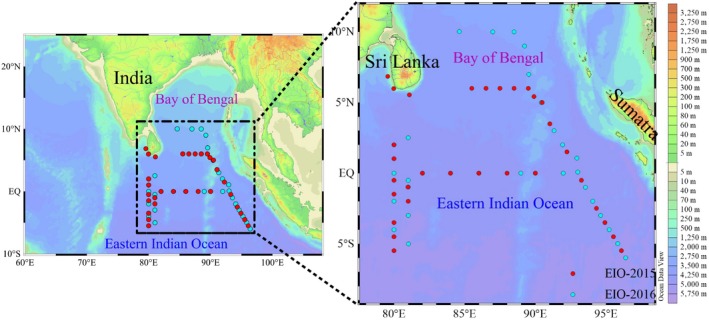
Study area and sampling stations during spring 2015 (red dots) and 2016 (blue dots)

Two cruises were conducted by the R/V *Shiyan‐1* in the EIO: March 15, 2015 to May 18, 2015 and March 20, 2016 to May 12, 2016, representing spring 2015 and 2016, respectively. Our study area extending from near‐shore coastal waters to oligotrophic open waters covered the entire EIO and its adjacent shelf, and 56 stations were investigated (Figure 1). At each station, seawater samples were collected from 7 depths within the upper 200 m water column using 12 L Niskin bottles equipped with a SeaBird CTD (Conductivity, Temperature, and Depth; SBE 19 Plus). Temperature, salinity, and depth were recorded in situ at the same time. Photosynthetically active radiation (PAR, 400–700 nm, µmol quanta m^−2^ s^−1^) was measured by an RBR sensor (XRX‐620).

Seawater samples for picophytoplankton were fixed with paraformaldehyde (1% final concentration) on board. To avoid loss of resolution and changes in cell counting due to fixation or freezing, FCM samples were kept in the dark without treatment at room temperature for 10–15 min, and then quickly freeze‐trapped in liquid nitrogen until analysis in the laboratory (Marie, Simon, Guillou, Partensky, & Vaulot, 2000). Seawater samples for nutrient analysis were filtered through 0.45 µm cellulose acetate membrane filters and then immediately refrigerated at −20°C for further analysis. Analyses for the determination of nutrient concentrations including ammonium, phosphate, nitrate, nitrite, and silicate were performed by a Technicon AA3 Auto‐Analyzer (Bran + Luebbe) according to the classical colorimetric methods. Dissolved inorganic nitrogen (DIN) defined as ammonium + nitrite + nitrate was analyzed using the copper‐cadmium column reduction method (Guo et al., 2014). Dissolved inorganic phosphorus (DIP) and silicate (DSi) were measured using spectrophotometry with standard molybdic acids and Murphy Riley molybdenum blue reagents according to Brzezinski and Nelson (1986) and Karl and Tien (1992), respectively.

Subsamples for size‐fractionated chlorophyll *a* (Chl *a*) analysis were filtered serially through 20 µm × 20 mm silk net, 2 µm × 20 mm nylon membrane, and 0.2 µm × 20 mm polycarbonate filters under a filtration vacuum of less than 100 mm Hg, then immediately refrigerated at −20°C. After returning to the laboratory, these filters were placed into 20 ml glass tubes, the pigments were then extracted by 5 ml 90% acetone, and quickly stored in the dark at 4°C for 24 hr. Finally, the Chl *a* contents were determined using a CE Turner Designs Fluorometer (Liu et al., 2016; Welschmeyer, 1994).

### 2.2 Flow cytometry analysis

**Figure 2:**
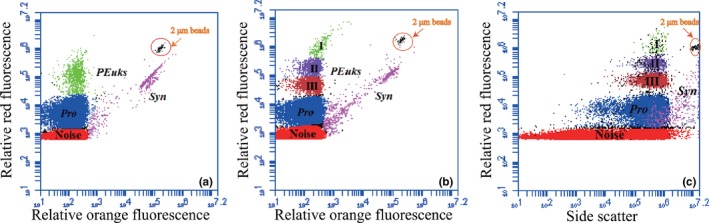
Flow cytometer (FCM) analysis of seawater samples collected from the eastern Indian Ocean (EIO)

Abundances of three picophytoplankton groups were enumerated using a flow cytometer (FCM, Becton‐Dickinson Accuri C6) equipped with a laser emitting at 488 nm. Data collection of all parameters was triggered by the 488 nm scatter signal. As a total volume of only 198 µl (flow rate at 66 µl/min running for 3 min) was analyzed, and the upper size limit for picoeukaryotes was usually 5 µm, above which the cells were very rare and could not be accurately quantified. Two µm fluorescent beads (Polysciences) were added as the instrument internal standard (Olson, Zettler, & DuRand, 1993). Different picophytoplankton populations were manually classified according to their amplitude, shape, and position of optical signals in the scatterplots of relative red fluorescence (FL3, >670 nm) versus relative orange fluorescence (FL2, 585 ± 42 nm) and FL3 versus side scatter (SSC). Three dominating populations including *Syn*, *Pro,* and *PEuks* were identified by FCM (Figure 2). Otherwise, *PEuks* in some special stations were also differentiated three subclusters (I‐III) by their distinct red fluorescence signals. However, it was difficult to identify and separate these subclusters from all stations, so that these several subclusters of *PEuks* would be combined when describing their abundance and distribution.

To avoid loss of dispersion and resolution of target cells in the scatterplot due to the overlapping signals with noise, the trigger threshold (~600) was set well below the lowest scatter signals from surface *Pro* cells. Spurious scatter events and fluorescence cross‐talk were minimized by setting the polarization of excitation laser perpendicular to the axis of flow. After these settings, 2 ml DI water was preferentially run at a steady flow rate of 66 µl/min to collect and gate the scatter and fluorescence signals of noise. If higher background counts were detected, the FCM was thoroughly cleaned with 5% bleach. The fluorescence and scatter signals were captured with user‐built detector assemblies with an extended range. However, these detectors cover a signal range of more than six decades by combining the signals from two photomultipliers that operate at different gains (Van et al., 2017). The relative gain settings were calibrated by a regression analysis of the events that fall within the linear window of both detectors. This approach can accurately detect all instantaneous picophytoplankton sizes ranging from dim surface dwelling *Pro* to bright *PEuks*.

## 3 RESULTS

### 3.1 Hydrological conditions

**Figure 3:**
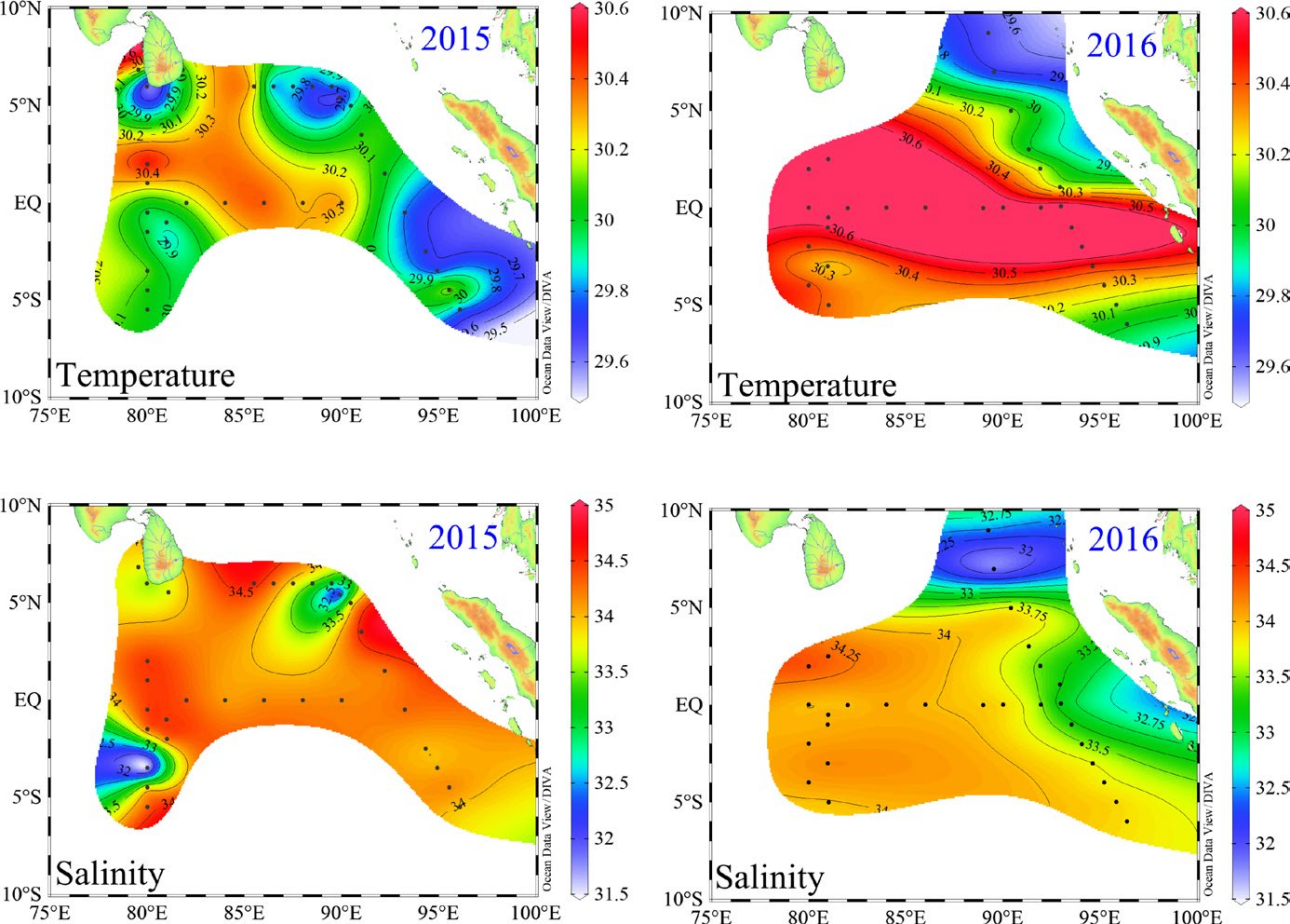
Spatial distributions of surface temperature (°C) and salinity in spring 2015 and 2016

**Figure 4:**
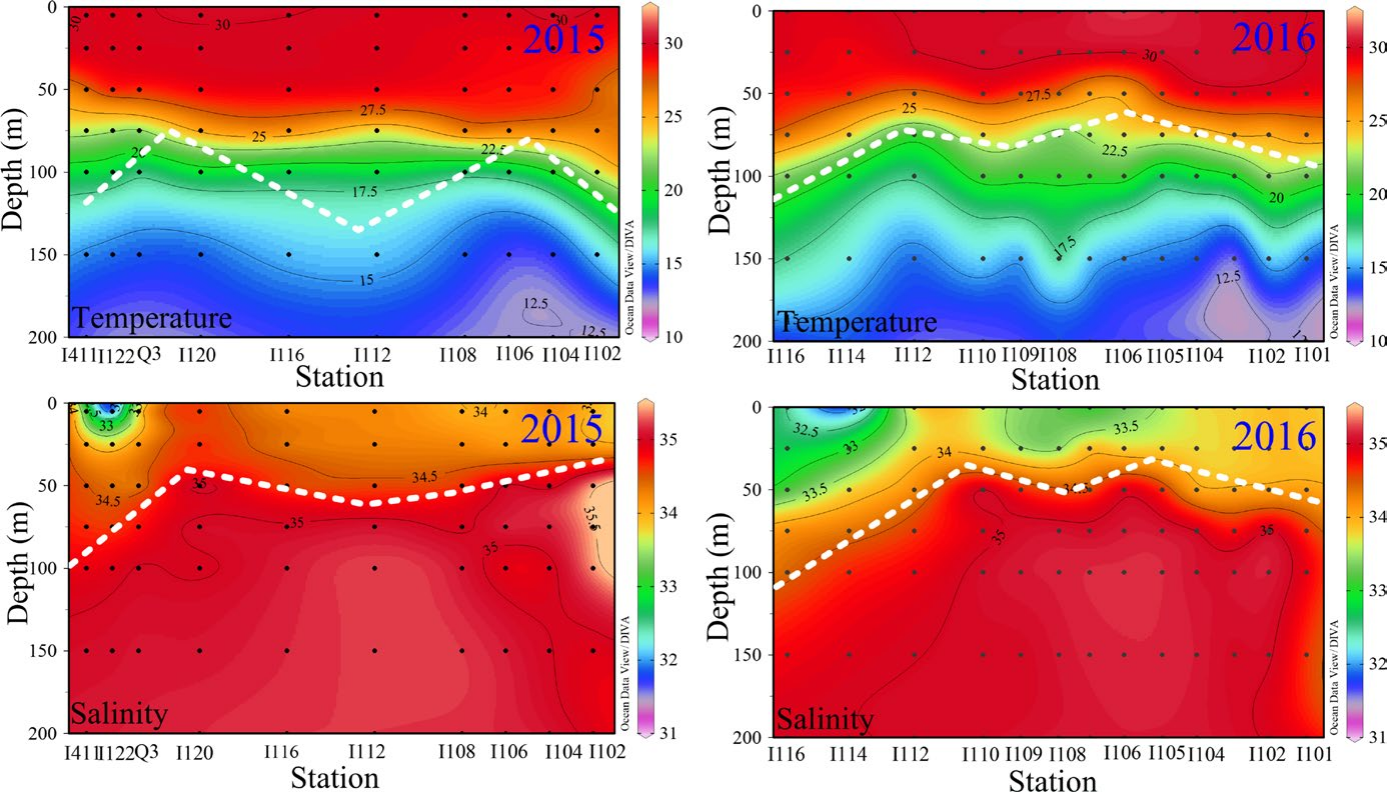
Vertical distributions of temperature (°C) and salinity along the Sumatra during spring 2015 and 2016

A surface warm tongue (>30°C) with salinity above 34 moved eastward from west along the equator during spring 2015 and 2016 (Figure 3), thereby indicating an apparent influence of the Wyrtki jets (WJ) (Wyrtki, 1973). The high levels of surface temperature and salinity were particularly observed between 80°E and 90°E around the equator. This was probably because the WJ was strongest between 60°E and 90°E and profoundly changed its water layer structure by removing the relatively high salinity and warm surface seawater from west and accumulating it in the east. The northeast part of the studied area represented a cold tongue with a low temperature of approximately 29°C, where was primarily influenced by the surface freshwater from the Bay of Bengal (BBR) (Sengupta, Bharath Raj, & Shenoi, 2006). Nevertheless, there were contrasting differences in physical background nearby the Sumatra between spring 2015 and 2016. Due to the strong coastal upwelling (see below Figure 4), surface temperature surrounding the Sumatra was below 29°C with a comparatively high salinity of approximately 34 during spring 2015. By contrast, surface seawater was of the large variations in both temperature and salinity during spring 2016, indicating that its hydrographic properties were potentially contributed by the combined effects of coastal upwelling and freshwater discharging from the coastal currents.

The vertical profiles of temperature and salinity along the Sumatra are shown in Figure 4. Water columns were apparently stratified during both years due to high surface temperature in spring. Shetye et al., (1993) reported that after the onset of the Southwest Monsoon, there were many indications of deep upwelling along the Sumatran coast, with subsurface isotherms and isopycnals tilting upward from depths of about 80–100 m within 40 km of the coast. However, the above‐reported deep upwelling along the Sumatran coast was fully applicable to our dataset. As such, apparent upwellings off the Sumatra were observed through the abnormal low temperature (<20°C at 75 m) and high salinity (>34 at 50 m) in the upper part of the water column (dashed white line).

**Figure 5:**
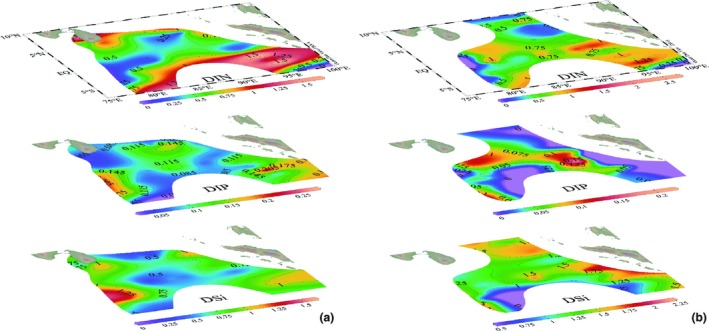
Horizontal distributions of DIN, DIP, and DSi (µmol/L) in the surface layers during spring (a) 2015 and (b) 2016

**Figure 6:**
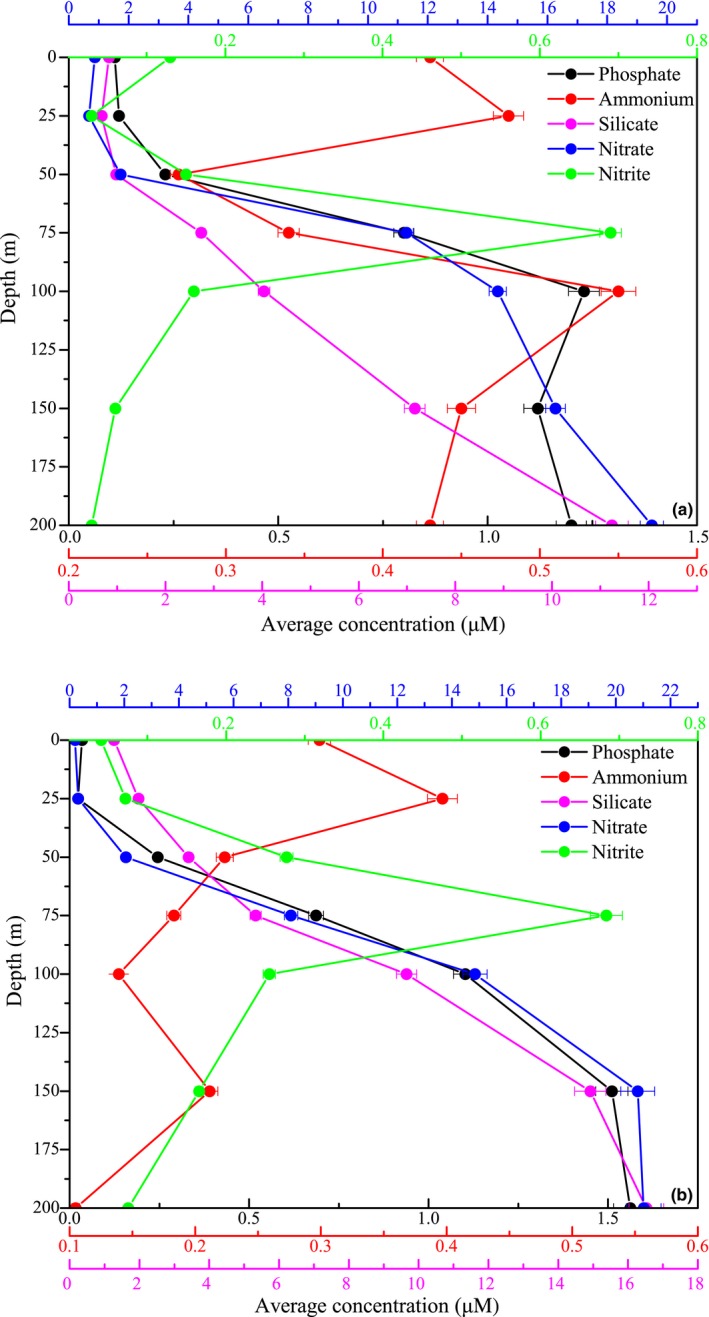
Vertical distributions of average nutrient concentrations with standard error over 0–200 m in spring (a) 2015 and (b) 2016

Due to pronounced stratification, surface nutrients (DIN, DIP, and DSi) were usually depleted during spring except for some coastal zones, where the freshwater discharged or coastal upwelling elevated the nutrient levels (Figure 5). Therefore, the average DIP, DSi, and nitrate concentrations appearing to follow a depth gradient with relatively higher levels at the bottom were apparently lower in the upper layer of the vertical patterns during spring 2015 and 2016 (Figure 6). Although low average nitrite concentrations were similarly observed at the surface layer (0–50 m) during both spring, they exhibited peak values (>0.65 µmol/L) around the subsurface 75 m, and then decreased sharply to <0.10 µmol/L near the bottom. Additionally, contrasting vertical patterns of the average ammonium concentrations from surface to bottom were observed between spring 2015 and 2016.

### 3.2 Comparisons of picophytoplankton abundance and chlorophyll *a* between spring 2015 and 2016

During spring 2015 and 2016, *Syn* were most abundant in the surface layers and only very few cells were counted below 100 m. Median values of *Syn *abundance in the surface layers reached 2.58 × 10^3^ and 6.13 × 10^3^ cells ml^−1^, respectively (Table 1). *Pro* dominated most regions of the EIO. The maximum abundance of *Pro* was >10^5^ cells ml^−1^, and median abundances of 0.55 × 10^4^ and 1.22 × 10^4^ cells ml^−1^ were recorded in the whole area during spring 2015 and 2016, respectively. *PEuks *were frequently absent at many open sea sites, and were much less abundant than *Syn* and *Pro*. Overall, *Pro* were approximately 1–2 orders of magnitude more abundant than *Syn* and *PEuks *in the EIO. In contrast to spring 2015, both *Syn* and *Pro* median abundances over the surface layers, water column integration, and whole area were dramatically higher in spring 2016. In the whole EIO, however, there was no obvious difference in median abundances of *PEuks *between spring 2015 and 2016.

**Table 1:** Range and median of picophytoplankton abundance (×10^3^ cells ml^−1^), PChl *a *concentration (µg L^−1^), and average percentage of PChl *a*/Total Chl *a*

Factors/Years	2015			2016		
	Surface layer	Water column integration	Whole area	Surface layer	Water column integration	Whole area
*Syn*						
Range	0.12–20.01	0.12–8.56	0.01–26.70	0.01–29.20	2.05–12.32	0.05–34.51
Median	2.58	1.29	0.94	6.13	3.85	3.45
*Pro*						
Range	0.57–37.18	0.89–53.20	0.52–208.00	0.32–83.76	9.59–53.23	0.32–225.30
Median	5.22	14.82	5.52	6.54	26.31	12.24
*PEuks*						
Range	0.01–2.14	0.01–4.52	0.01–16.60	0.01–2.34	0.36–2.22	0.01–13.26
Median	0.58	0.83	0.33	0.28	1.03	0.33
PChl *a*						
Range	0.014–0.194	0.034–0.233	0.004–0.448	0.016–0.312	0.068–0.206	0.001–0.506
Median	0.063	0.096	0.074	0.116	0.135	0.122
Percentage (%)	68.4	52.4	49.6	63.5	48.3	44.9

Picophytoplankton Chl *a* concentrations (PChl *a*) were significantly higher in spring 2016 than in spring 2015 (Table 1). Notwithstanding PChl *a* concentrations showed considerably low values throughout the EIO, they are responsible for a large fraction of the total Chl *a *concentrations, accounting for average percentage 49.6% and 44.9% of PChl *a*/Total Chl *a* in the whole EIO, respectively. Very similar temporal variations were found between PChl *a* concentrations and *Syn* and *Pro* abundances.

### 3.3 Horizontal distributions of picophytoplankton abundance and chlorophyll *a*

**Figure 7:**
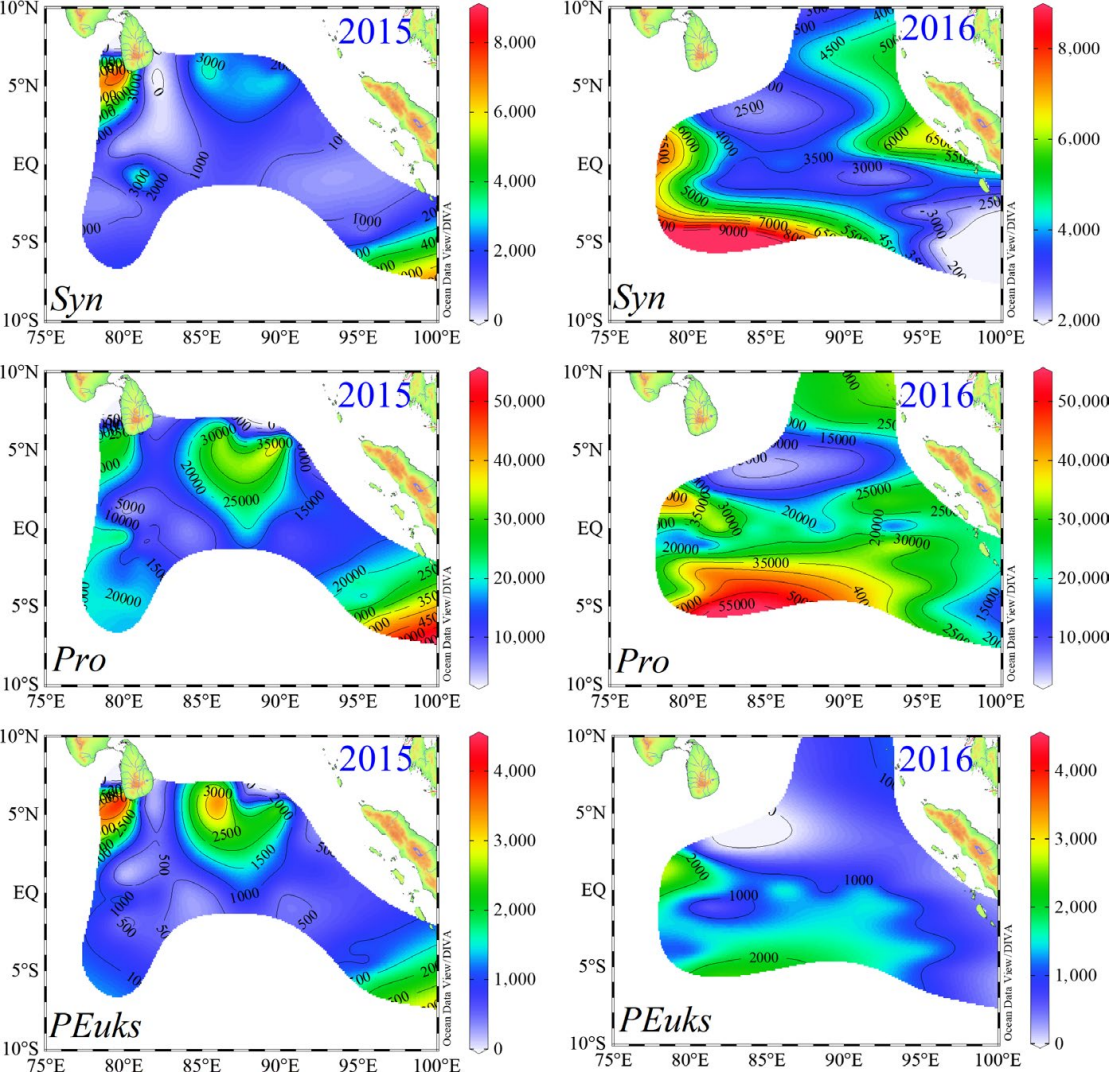
Horizontal distributions of water column vertically integrated abundances of *Syn*, *Pro *and *PEuks* (cells ml^−1^)

Since *Pro* abundance was seldom observed at the surface layer, the water column integrated abundance involving a series of abundance variations with depth was better suited for spatial distributions of picophytoplankton than the averaging through the sampled layer. The horizontal distributions of water column integrated abundance over top 200 m for three picophytoplankton populations are illustrated in Figure 7. During spring 2015, high abundance of *Syn* was mostly encountered around the Sri Lanka island. A relatively high value of *Syn *abundance was observed roughly along the coast of Sumatra, in which *Syn* were predominantly affected by the coastal upwelling (Figures 3 and 4). *Pro* were the numerically dominant component of picophytoplankton communities in the EIO, surprisingly, they presented the highest abundance in the coastal upwelling zones nearby the Sumatra. In a pattern that was similar to that observed for *Syn*, *PEuks *were also found in high abundance surrounding the coastal zones of Sri Lanka island and Sumatra, decreasing gradually from the coastal zones to the open ocean. In spring 2016, the maximal *Syn* abundance exceptionally concentrated to the open sea of the southern equator, particularly within areas influenced by the Wyrtki jets. The sub‐high values of *Syn* abundance were primarily distributed around the Sumatra due to coastal upwelling and freshwater influence. However, *Pro* remained ubiquitously in the whole survey area, and were similarly abundant in the southern region along the equator. Abundance of *PEuks* had a similar distribution pattern as *Syn*, with high values appearing in the southwest of study area. Overall, there were no coherent patterns between the spring 2015 and 2016 in the spatial distributions of picophytoplankton.

### 3.4 Vertical distributions of picophytoplankton abundance and chlorophyll *a*

**Figure 8:**
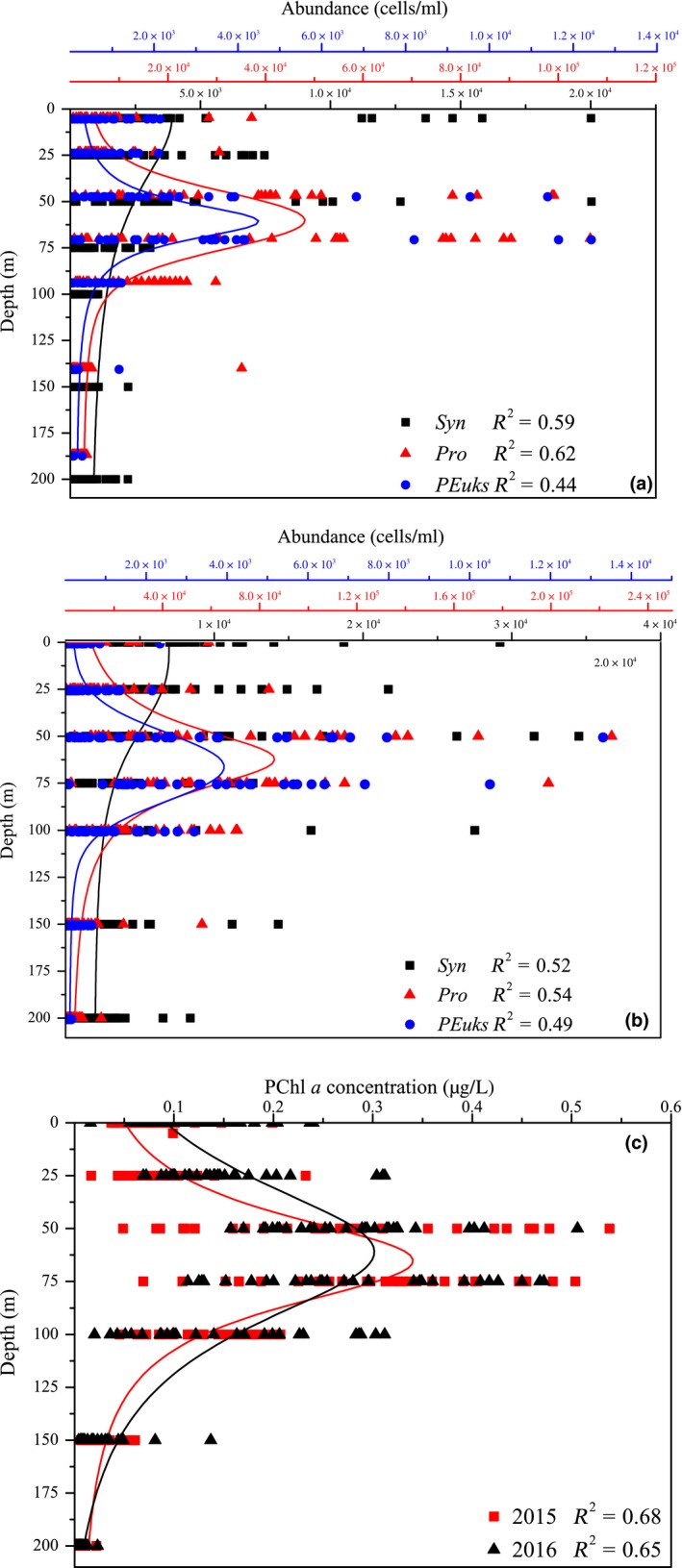
Vertical profiles of *Syn*, *Pro*,* PEuks* abundances, and PChl *a *concentrations during spring 2015 (a) and 2016 (b)

To clearly understand and compare the vertical pattern, all the data points of picophytoplankton abundance and PChl *a *concentration against depth were analyzed to fit the nonlinear curves (Figure 8). This nonlinear analysis provided the real shape of the response curve of abundance to depth and highlighted the variance (*R* ^2^) in regression models. There were striking similarities between the two years in vertical distribution of three picophytoplankton groups. For *Syn*, the maximum abundance distinctly occurred in the surface (0–50 m), and then decreased gradually with increasing depth. In contrast, *Pro* abundance exhibited a significant difference in vertical distribution, and typically displayed the maximum at the subsurface (50–75 m), but decreased rapidly down to the minimum by 150–200 m. *PEuks* and *Pro *had similar vertical patterns, with the maximum abundance occurring at the subsurface. Nonetheless, *PEuks* were less important numerically than *Pro*, and which were almost one order of magnitude lower than *Pro*.

The depths of the deep PChl *a* maximum layer (DPCM) were extremely similar between spring 2015 and 2016, which both occurred approximately at the subsurface. By contrast, the subsurface peaks in *Pro* and *PEuks* abundances occurred closely to the DPCM. However, the depths between the maximum abundance of *Pro* and *PEuks *and the DPCM were fairly similar.

### 3.5 Relationship between picophytoplankton abundance and biological and environmental factors

In both spring, abundances of *Syn*, *Pro,* and *PEuks* were positively correlated with each other (Pearson rank correlation coefficient *r > *0.46, *p* < 0.001). Furthermore, *Pro* and *PEuks* abundances were also positively correlated with PChl *a* concentration (*r > *0.19, *p* < 0.001). Differently, *Syn *abundance was positively correlated with PChl *a* concentration in spring 2016 (*r = *0.64, *p* < 0.001), while no significant correlation was found in spring 2015 (Table 2).

**Table 2:** Pearson's rank correlation coefficient between picophytoplankton and PChl *a* concentration

Year	Factor	*Syn*	*Pro*	*PEuks*	PChl *a*
	*Syn*		0.517**	0.608**	0.112
2015	*Pro*	0.517**		0.853**	0.648**
	*PEuks*	0.608**	0.853**		0.565**
	*Syn*		0.858**	0.455**	0.642**
2016	*Pro*	0.858**		0.532**	0.652**
	*PEuks*	0.455**	0.532**		0.195**

Note

*Syn*, *Pro* and *PEuks* abundances were log transformed prior to analysis.

** Correlation is significant at the 0.01 level (2‐tailed).

Canonical correspondence analysis (CCA) is a popular multivariate extension analysis of weighted averaging ordination, particularly developed to relate biological assemblages of species to known variation in the environment (Braak, 1986; Braak & Verdonschot, 1995). Questions in biological ecology that have typically been studied by indirect gradient analysis can be answered more directly by the CCA. In addition, the CCA is an efficient ordination analysis when species have regression response curves or surface (vertical) with respect to environmental gradients, and is therefore more appropriate for analyzing data on species and environmental variables than other analysis (Braak, 1986; Hill, 1991). In summary, picophytoplankton variation can be directly related to environmental variation by the CCA analysis.

**Figure 9:**
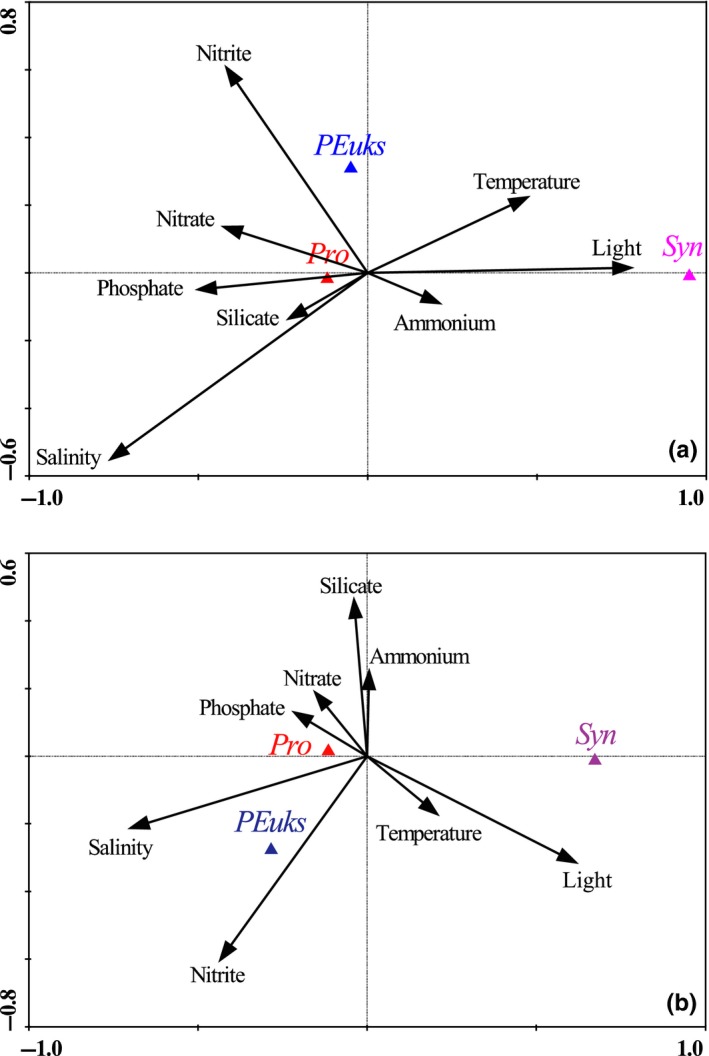
Canonical correspondence analysis (CCA) ordination diagram of three picophytoplankton groups with environmental variables during spring (a) 2015 and (b) 2016 in the eastern Indian Ocean (EIO)

The CCA leads to an ordination diagram in which point represents species, and vector represents environmental factor (Figure 9). The species‐environment correlation coefficients of CCA axes 1 and 2 in spring 2015 were 0.914 and 0.748, respectively, while they were 0.964 and 0.896 during spring 2016. The *p*‐values for the test of significance of all canonical axes during both spring were <0.003. The correlation coefficients among environmental axes were 0, and the species axes were approximately vertical to each other. These statistical tests collectively indicated that such a diagram shows the variable patterns of picophytoplankton that could be explained best by the environmental variables. In the two diagrams, the length of arrows of nitrite, salinity, and light was relatively long during both spring, indicating they were the primary variables affecting the variation of picophytoplankton. *Pro* closed to species axis were intimately related to environmental variables, but nutrients were especially significant. For example, *Pro* showed clearly correlation with DIP, DSi, and nitrate in spring 2015, with the correlation coefficients of 0.962, 0.816, and 0.897, respectively, suggesting that nutrient availability played an important role in controlling the variable patterns of *Pro*. In particular, *Pro* abundance showed a strong correlation with DIP (Pearson rank correlation coefficient *r* > 0.39, *p* < 0.001; *n* = 382), indicating that the variation of *Pro* was profoundly affected by the DIP concentration. A significant link between *PEuks* and nitrite was found during both spring (*r* > 0.23, *p* < 0.001), indicating nitrite among nutrients was a key factor in regulating *PEuks* abundance. *Syn* abundance did not show a clear correlation with nutrients; thus, the variability in nutrients seemed to have little effect on the *Syn* variation. However, *Syn* abundance was strongly related to temperature (*r* > 0.39, *p* < 0.001) and light (*r* > 0.36, *p* < 0.001), suggesting the growth of *Syn* had apparent requirement for the temperature and irradiance.

## 4 DISCUSSION

### 4.1 Characteristics of picophytoplankton abundance

Remarkable differences in *Syn* and *Pro* abundances were observed between spring 2015 and 2016, which showed these picocyanobacteria were approximately 2 ~ times higher abundances in spring 2016 (Table 1). As in spring 2015, the maximal *Syn* and *Pro *abundances were only present in the coastal zones. In addition to the coastal zones, maximum *Syn* and *Pro *abundances exceptionally appeared around the southern equator during spring 2016 (Figure 7). These observations led us to speculate that the partial increase in abundances of *Syn* and *Pro *along the southern equator makes them overall show more abundance in spring 2016. *PEuks* displayed considerably high abundance nearby the coastal zones (particularly around the Sri Lanka island) during spring 2015, while they were found in high abundance surrounding the southwest of study area in spring 2016. Although *PEuks* abundances in two years have different spatial distributions, no contrasting differences in abundance of *PEuks *were observed between spring 2015 and 2016, thus suggesting that the impacts of different local versus oceanographic conditions on the abundance variability can effectively counterbalance the interannual difference of *PEuks *abundance.

According to our correlation analysis, PChl *a* concentration correlated positively with the abundances of three picophytoplankton groups in both spring (*r* > 0.19, *p* < 0.001), with the exception of *Syn *in spring 2015 (Table 2). This indicates that *Pro* and *PEuks* are the most important variables controlling the PChl *a *concentration of spring 2015, while the PChl *a *concentration of spring 2016 is collectively supported by the three picophytoplankton groups. Thus, the temporal differences were observed for the PChl *a *concentration as a consequence of interannual difference in abundances of three picophytoplankton groups.

### 4.2 Characteristics of picophytoplankton horizontal distribution

**Figure 10:**
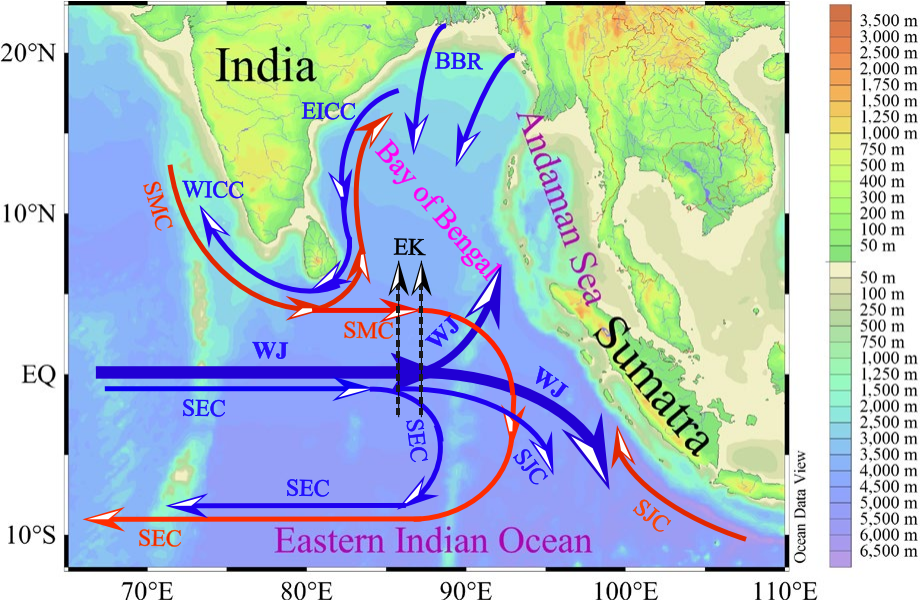
A schematic showing the circulations and water masses in the eastern Indian Ocean (EIO) (after Wyrtki, 1973; Shenoi, Saji, & Almeida, 1999; Peng et al., 2015). BBR: Bay of Bengal Runoffs; EICC: East India Coastal Current; EK: Ekman transports; SEC: South Equatorial Countercurrent; SJC: South Java Current; SMC: Northeast and Southwest Monsoon Currents; WICC: West India Coastal Current; WJ: Wyrtki jets. Currents occurring in summer (winter) were colored red (blue), while the WJ occurred during spring and fall. The thickness of the curve represented the relative magnitude of the current

It is well known that the variation in temperature and salinity (T‐S) is a useful aid in discriminating the various water bodies and studying their sources and mixing (Tomczak, 1999). Combined with our spring data of water temperature and salinity, the possible vital water masses were accurately discriminated, such as the Wyrtki jets, coastal current from Bay of Bengal and upwelling along the Sumatra (Figures 3 and 4). However, only our T‐S dataset is not yet fine enough to fully explain the changes in circulations throughout the EIO. Therefore, we supplemented the circulation system based on the references and summarized a intuitive schematic of circulations or water masses in the EIO (Figure 10). Sri Lanka island is a significant place encircled by the Indian Ocean, and is considered as a center of import and export commercial harbors. Most of coastal waters around the Sri Lanka island are influenced by the increases of pollution and eutrophication (Silva, 2007). Simultaneously, the East India Coastal Current (EICC) along the western boundary of the Bay of Bengal flows equatorward and bifurcates east of the Sri Lanka island, but one bifurcation of its source waters characterized by nutrient enrichment continues along the coast of Sri Lanka island (Vinayachandran et al., 2005). This studied coastal area is relatively abundant in nutrients and is therefore suitable for picophytoplankton development (Figure 5). This increase in nutrients near the coast can induce the regional increase in abundance and PChl *a* concentration, however, which is not yet fine enough to fully represent the total PChl *a* concentration throughout the EIO. The total PChl *a* concentration in the EIO remained lower than other oceans. *Syn* can be subdivided into open ocean and coastal phylogenetic clusters which have not salt requirements for growth (Dufresne et al., 2008; Sohm et al., 2015). Our CCA analysis revealed that *Syn* abundance was negatively correlated with nutrients and salinity (*r < −*0.26, *p* < 0.001) in both spring (Figure 9), suggesting *Syn* concentrating in the coastal waters are primarily composed of the coastal phylogenetic cluster. Although negative correlation in CCA analysis, at times, is just a superficial presentation for the relationship between biotic and abiotic factors, it indicated nutrient supply is not the key limiting factor for *Syn* growth in the EIO. In situ as well as experimental observations showed that nutrients and its efficient uptake significantly influence the growth of *Syn* (Bemal & Anil, 2016). *Syn* also exhibit a wide range of elemental stoichiometry, including carbon‐to‐nitrogen ratios and increased their carbon‐to‐phosphorus ratios in response to low dissolved phosphorus availability (Baer et al., 2017). The higher growth rate of coastal strains than the open ocean strains is mainly attributed to differences in nutrient concentrations between these two regions (Liu et al., 1998; Sohm et al., 2015). As such, *Syn* were generally the predominant groups in coastal waters of the Sri Lanka island attributing to a combined result of different taxonomic composition and superior ability to adapt a wide variety of nutrient concentrations. Previous studies revealed that *PEuks* are most abundant in nutrient‐rich than in oligotrophic oceans (Wang, Huang, Liu, & Chen, 2014; Worden & Not, 2008). Indeed, nutrient availability had pronounced effects on *PEuks *abundance according to our CCA analysis. Such anthropogenic loading contaminants and freshwater discharging from the EICC with high nutrients in coastal waters of the Sri Lanka island contributed to the fairly high abundance of *PEuks*. Surface temperature surrounding the Sumatra was below 29°C with a high salinity of approximately 34 during spring 2015 and 2016 (Figure 3), indicating that its hydrographic properties are prominently attributed to the coastal upwelling. Overall, the co‐occurrence of *Syn* and *PEuks* in the coastal upwelling zones along the Sumatra are not surprising given the existence of numerous subclusters and the versatility of them had been related to their ability to adapt a wide variety of biogeochemical conditions.

In general, *Pro* dominate in the oligotrophic environments of subtropical and tropical oceans, but their distributions are limited at high latitudes by low temperature (Johnson et al., 2006) and are also limited in coastal waters, upwelling areas and temperate oceans by environmental factors such as nutrients availability, heavy metal toxicity, and competition among groups (Lee, Choi, Youn, & Roh, 2014). No significant correlations were found between *Pro* abundance and temperature in both spring (Figure 9), thus implying temperature is not the most important factor controlling the horizontal distribution of *Pro* in the tropical EIO. However, low temperature has significant limiting effect on the vertical distribution of *Pro* (*r* > 0.23, *p* < 0.001). Interestingly, *Pro* exhibited a remarkably high density in the coastal zones nearby the Sumatra and Sri Lanka island, while this finding was also observed in the Pearl River Estuary where an intrusion of oligotrophic seawater enriched *Pro* abundance (Zhang et al., 2013). Within this study, *Pro*'s persistent or sudden appearance nearby the nutrient‐rich coastal waters or upwelling zones could not be simply associated with the intermittent intrusion phenomenon. Based on our CCA analysis, the close associations between nutrients and *Pro* abundance suggested that nutrient availability was the crucial variable in controlling the *Pro *distribution, especially for the DIP limitation (<0.3 µmol/L at the surface). Baer et al., (2017) recently revealed natural populations of *Pro* allows for a broad range of cellular carbon‐ and nitrogen‐to‐phosphorus ratios under low nutrient stress. Moreover, some *Pro *can adapt to the coastal environments because of the capacity to absorb and utilize amino acids, glucose, and dimethylsulfoniopropionate (Gómez‐Baena et al., 2008; Vila‐Costa et al., 2006). Although the limited effects of high concentrations of nutrients and heavy metals in coastal regions or upwelling zones could constrain *Pro* growth, the increase in nutrient availability particularly in the oligotrophic marine systems can appreciably elevate the *Pro* abundance. In addition, Johnson et al., (2006) also presented that nutrients appear to play a vital role in shaping different *Pro* distributions in the oligotrophic Atlantic Ocean. However, further studies are obviously necessary to shed light on the possible mechanism of the increase of *Pro* abundance in these coastal waters or upwelling zones.

Waters of very low salinity and temperature (32 and 29°C, respectively) were presented in the northern Bay of Bengal, thereby indicating they are profoundly influenced by freshwater from the Bay of Bengal runoffs (BBR). The BBR are typically in high nutrients since they are generated through the combined effects of riverine discharge, excess precipitation and contamination. Subsequently, these nutrient‐rich waters spread widely around the northern Bay of Bengal (Sengupta et al., 2006). The aforementioned study showed that *Pro* are not strictly restricted to the oligotrophic part of open oceans and prefer warm mesotrophic conditions as well (i.e., waters with a detectable level of nutrients). Accordingly, slightly high abundances of three picophytoplankton groups co‐occurred in the southern Bay of Bengal because they all can benefit from the increased nutrient availability carried by the BBR.

Preliminary results showed picophytoplankton were exceptionally abundant along the equator during spring 2016 (Figure 7). However, their abnormal appearance along the equator appears to be associated with the annual changes of the circulations or water masses. Surface water structure around the equator was profoundly modulated by the WJ through removing the relatively high salinity (above 34) and warm (>30°C) surface seawater from west and accumulating it in the east (Figures 3 and 4). The possibility of exchange in seawater is essential in explaining the spatial variations in picophytoplankton abundances along the equator. Hence, the abnormal presence of picophytoplankton in spring 2016 along the equator was presumably dependent on the advection processes supported by the WJ. Altogether, as a result of differential responses of three picophytoplankton groups to the changes in physical and chemical environments induced by the annually variable circulations and water masses, we have observed very different patterns of these three groups between spring 2015 and 2016 in their spatial distribution.

### 4.3 Characteristics of picophytoplankton vertical distribution

**Figure 11:**
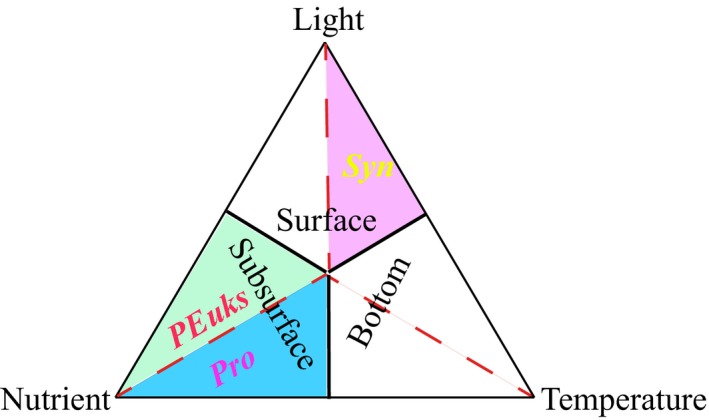
Schematic of the environmental factors governing the vertical distribution of picophytoplankton in the eastern Indian Ocean (EIO). Surface, 0–50 m; Subsurface, 50–75 m; Bottom, 100–200 m

A simple conceptual model for vertical patterns of picophytoplankton based upon environmental parameters is proposed (Figure 11). Three picophytoplankton groups showed two different characteristics of vertical distribution in both spring. One group, including *Syn*, was frequently abundant in the surface and then gradually decreased with depth. Another group, including *Pro* and *PEuks*, was characterized by the subsurface maximum (Figure 8). Jiao, Yang, Koshikawaz, and Watanabez (2002) stated that the vertical distribution of *Syn* can be understood in terms of nutrient and light availability. In the present study, however, temperature and light irradiation tended to be the factors mostly affecting *Syn* abundance and distribution. This finding well agreed with previous temperature analysis on diverse strains of *Syn* reported by Mackey et al., (2013) and Sohm et al., (2015), who presented *Syn *have evolved a suite of temperature acclimation strategies to underlie the larger geographic range of this group. In addition to the influence of temperature, Grébert et al., (2018) recently demonstrated that light is a key ambient parameter in shaping the global ocean distribution and community structure of *Syn*. Furthermore, Moore, Goericke, and Chisholm (1995) stated the restriction of *Syn* to euphotic layer may be the result of light limitation imposed by its efficient accessory pigments. The euphotic depths vary greatly among stations, but are deeply modified by the transparency induced by fresh water inputs, indicating the variability in the transparency is an important factor affecting the distribution of *Syn*. However, this phenomenon occurs frequently in the estuaries where the low transparency is a key limiting factor for the growth of *Syn* (Callieri & Cristiana, 2008; Lee et al., 2014; Somogyi, Felföldi, Dinka, & Vörös, 2010; Zhang et al., 2013). Our study areas are mostly far away from the estuaries (only 3 stations near the Sri lanka during spring 2015), thus the low transparency (i.e., the euphotic layer thickness) affected by fresh water inputs appears to have little influence on the vertical distribution of *Syn*. Altogether, *Syn* were mainly restricted to the upper waters and generally exhibited a clear vertical decrease with depth as temperature and light availability became more significant than nutrient availability. On the contrary, *Pro* and *PEuks *abundances showed strong positive correlations with nutrients, indicating they are mostly profited from the environment in condition with high nutrients, hence nutrients may be the most probable factors to play a role in vertical distributions of *Pro* and *PEuks* in the EIO. DuRand, Olson, and Chisholm (2001) reported the depth of *Pro* maximum abundance is significantly correlated with the nutricline depth. Indeed, the nutricline in the EIO was near the subsurface 50–75 m during both spring (Figure 6). Meanwhile, Moore et al., (1995) proposed the growth of most *Pro* strains is inhibited at temperatures higher than 25°C. The temperature of surface seawater in the EIO was fairly high with an average value of approximately 30°C (Figure 3). Therefore, the high temperature at the surface potentially limited the *Pro* growth. Although not statistically significant in the CCA analyses, too low temperature similarly has limiting effect on the vertical distribution of *Pro* as discussed above (*r* > 0.23, *p* < 0.001). Malmstrom et al., (2010) similarly observed *Pro* ecotypes each respond differently to the variation in temperature and light. The temperature may be another reason for the vertical variation of *Pro* abundance in the EIO. Consequently, *Pro* with a maximum abundance near the subsurface were probably formed by the combined effects of temperature and nutrient availability in the EIO. *PEuks* were less light dependent and could thrive over a very wide light gradient resulting from their ecophysiological heterogeneity (Worden & Not, 2008). Previous studies implied that *PEuks* are ubiquitous in the marine environment with population maximum occurring frequently in low irradiance, but high nutrient environments (Zhang et al., 2013). In particular, *PEuks* are able to tolerate lower temperature. There were thus high abundance of *PEuks* evidently at 50–75 m depth (which did not extend to the surface).

## AUTHOR CONTRIBUTIONS

J Sun designed this study. YW, GZ, JC, JW, CD, and XZ performed the experiments and analysis. YW wrote the manuscript and prepared the tables and figures. All authors edited the manuscript. No conflict of interest exits in the submission of this manuscript, and manuscript is finally approved by all authors for publication.

## Data Availability

Data and computer codes for analyses are available in GitHub repository: https://github.com/Picophytoplankton/Ecology-and-Evolution.
